# Toll-like receptors 4 and 9 are responsible for the maintenance of the inflammatory reaction in canine steroid-responsive meningitis-arteritis, a large animal model for neutrophilic meningitis

**DOI:** 10.1186/1742-2094-9-226

**Published:** 2012-09-27

**Authors:** Arianna Maiolini, Regina Carlson, Andrea Tipold

**Affiliations:** 1 Department of Small Animal Medicine and Surgery, University of Veterinary Medicine Hannover, Buenteweg 9, D-30559, Hannover, Germany; 2 Center for Systems Neuroscience, University of Veterinary Medicine Hannover, Buenteweg 17, D-30559, Hannover, Germany

## Abstract

**Background:**

Steroid-responsive meningitis-arteritis (SRMA) is a systemic inflammatory disease affecting young adult dogs and a potential large animal model for neutrophilic meningitis. Similarities between SRMA and infectious central nervous system (CNS) diseases in lymphocyte subsets suggest an infectious origin.

Toll-like receptors (TLRs) are pattern recognition receptors playing an important role in innate immunity. Due to their ability to recognize both self and non-self antigens, we hypothesize that TLRs are among the key factors for the induction of the inflammatory process in SRMA and provide an indirect hint on the etiology of the disease.

**Methods:**

The expression profile of cell surface TLRs (TLR2, TLR4 and TLR5) and intracellular TLRs (TLR3 and TLR9) of canine leukocytes was analyzed by immunophenotyping and subsequent flow cytometric measurements. Experiments were performed on cerebrospinal fluid (CSF) and peripheral blood (PB) samples of dogs affected with SRMA during the acute phase (n = 14) as well as during treatment (n = 23) and compared with those of dogs with bacterial meningitis (n = 3), meningoencephalitis of unknown etiology (n = 6), neoplasia of the central nervous system (n = 6) and a group of dogs with miscellaneous neurological diseases (n = 9). Two additional control groups consisted of dogs with pyogenic infections (n = 13) and of healthy dogs (n = 6).

**Results:**

All examined groups showed a high percentage of TLR2, TLR4 and TLR5 positive PB polymorphonuclear cells (PMNs) in comparison to healthy dogs. Very high values of TLR9 positive PB PMNs were detected in acute SRMA. Only a few similarities were found between SRMA patients and dogs with pyogenic infections, both groups were characterized by high expression of TLR4 positive PB monocytes. Glucocorticosteroid therapy reduced TLR2, TLR4 and TLR9 expression in PB monocytes.

**Conclusions:**

A relatively high expression of TLR4 and TLR9 in acute SRMA suggests that these two receptors might be involved in the inflammatory process in SRMA, enhancing the autoimmune reaction. Systematic CSF cell analysis for TLRs can be performed in future treatment studies in larger animals, such as dogs.

## Background

Steroid-responsive meningitis-arteritis (SRMA) is a systemic inflammatory disease affecting young adult dogs. It is the most common cause of meningitis [1] and the most common cause of fever of unknown origin in dogs [2]. In recent years SRMA has become well-recognized in veterinary practice, although a deep understanding of the disease is still lacking. Similarities between SRMA and infectious central nervous system (CNS) diseases in lymphocyte subsets suggest that the immune response in SRMA might be triggered by an antigen [3]. However, such infectious agents were not directly detected [4]. SRMA has been proposed to be a potential large animal model for Kawasaki disease [5], especially since systematic flow cytometric (FACS) analysis of CSF is feasible in larger animals, such as dogs [6].

Toll-like receptors (TLRs) are pattern recognition receptors which recognize both invading pathogens (through pathogen-associated molecular patterns, PAMPs) and endogenous molecules produced by injured tissue (through damage-associated molecular patterns, DAMPs) [7].

This recognition process plays a role in innate immunity and in the development of the adaptive immune response [8,9]. Additionally, TLRs may be involved in the induction of chronic inflammation and autoimmune reactions [9-12]. There are many examples of systemic human diseases in which an association with TLRs has been found [13], including systemic lupus erythematosus [14], giant cell arteritis [15,16], Sjögren’s syndrome [17], autoimmune arthritis [18] and multiple sclerosis [19]. In dogs, TLRs have been found up-regulated in inflammatory bowel disease [20]. The TLR expression on CSF leukocytes has not yet been widely studied.

To date, SRMA is believed to be characterized by a Th2-mediated immune response [21], but it is still unclear if this reaction is triggered by environmental factors or self-antigen (hit-and-run principle).

Due to their ability to recognize both self (DAMPs) and non-self (PAMPS) molecules, TLRs are suspected to be involved in the inflammatory process in SRMA. To confirm the hypothesis that SRMA is triggered by an environmental factor, such as a bacterial infection, which is specifically changing the TLR pattern, the expression profile of cell surface TLRs (TLR2, TLR4 and TLR5) and intracellular TLRs (TLR3 and TLR9) were examined on canine leukocytes. An indirect hint on the etiology of SRMA was expected.

## Methods

### Dog population and samples

The study population consisted of 80 dogs referred to the Department of Small Animal Medicine and Surgery, University of Veterinary Medicine, Hannover, Germany between May 2009 and April 2011. The studies were conducted according to the ethical guidelines of the University for Veterinary Medicine Hannover. Depending on the clinical diagnosis, the dogs were assigned to one of the following groups (see Table 1).

**Table 1:** Distribution of disease categories

**Diseases**	**Findings**	**Number of dogs**
SRMA Acute (SRMA A)	Dogs with fever, cervical pain, neutrophilic leukocytosis and pleocytosis, no pre-treatment with glucocorticosteroid	14
SRMA Therapy (SRMA Th)	Dogs from SRMA A group, asymptomatic under long-term glucocorticosteroid treatment	23
Bacterial meningitis (BM)	Dogs with meningitis/meningoencephalitis caused by bacterial infections	3
Meningoencephalitis of unknown etiology (MUE)	Dogs with clinical, CSF, MRI and/or pathological findings consistent with meningoencephalitis, in which no causative agent has been identified.	6
Neoplasia (Neopl.)	Dogs with clinical, CSF, MRI and/or pathological findings consistent with neoplasia of the CNS	6
Miscellaneous (Mix)	Dogs with miscellaneous non-inflammatory neurological diseases including intervertebral disc disease, peripheral nervous system diseases and idiopathic epilepsy	9
Pyogenic infection (Pyo)	Dogs suffering from diseases caused by pyogenic infections, such as pyometra, pyothorax and bacterial peritonitis	13
Healthy	Healthy dogs	6

SRMA Acute (SRMA A): The diagnosis of SRMA was supported by the detection of typical findings during physical and neurological examinations, complete blood and CSF examinations, cervical radiographs, elevated IgA levels in CSF and serum and the absence of other conditions causing cervical pain [22]. Dogs with the acute form of SRMA, but pretreated with glucocorticosteroids prior to CSF puncture were excluded from the study.

SRMA Therapy (SRMA Th): dogs from the former group under glucocorticosteroid treatment that did not show clinical signs at the time of sampling. Dogs under treatment for SRMA received prednisolone, with dosages ranging from 1 mg/kg/24 h to 0.5 mg/kg/48 h.

The other groups were: bacterial meningitis or meningoencephalitis (BM); meningoencephalitis of unknown etiology (MUE); CNS neoplasia (Neopl) and a group of dogs with miscellaneous neurological diseases (Mix) (see Table 1). In dogs with BM, MUE, CNS neoplasia and dogs with miscellaneous neurological diseases, in addition to the diagnostic procedures described for SRMA, magnetic resonance imaging (MRI), electrophysiological studies, surgery and histopathology contributed to the diagnosis.

Since SRMA is considered to be a systemic inflammatory disorder [23], leukocytes from dogs with pyogenic infections not affecting the nervous system (Pyo) were evaluated as a further control group. Another control group (Healthy) consisted of privately owned blood donors from the hospital and were considered to be healthy because history, complete physical examination, blood examination and clinical follow-up examinations did not reveal any abnormalities. The owners approved the blood examinations.

From each dog five mL of blood were collected via cephalic or saphenous venipuncture into tubes containing ethylene diamine tetraacetic acid (EDTA) for collection of peripheral blood (PB) leukocytes.

Cisternal cerebrospinal fluid (CSF) collection under general anesthesia was part of the work-up in all dogs for collection of CSF leukocytes with the exception of healthy animals and patients with pyogenic infections.

### Isolation, permeabilization and fixation of peripheral blood leukocytes

After collection, 1 mL of EDTA PB was used for staining of cell surface TLRs (TLR2, TLR4 and TLR5) and 0.5 mL for intracellular staining (TLR3 and TLR9). Leukocytes were fixed, in order to preserve their marker expression.

For the staining of cell surface TLRs, the cells were fixed using a previously described method of preparing blood leukocytes for flow cytometric analysis [24]. The method has been previously validated from Burgener and Jungi [25] for detection of TLRs on canine leukocytes. Briefly, the blood was mixed with the same volume of preheated 0.4% formaldehyde (diluted in phosphate buffered saline (PBS; containing 137 mM sodium chloride, 2.7 mM potassium chloride, 8.1 mM disodium hydrogen phosphate, 1.5 mM monopotassium phosphate, pH 7.4)) and incubated for four minutes at 37°C. Successively, 40 mL of warmed lysing buffer (0.83% ammonium chloride/0.01 M Tris chloride, pH 7.4) was added and the mixture was incubated at 37°C until red-cell lysis was observed (about one to two minutes). After centrifugation at 160 x g for 10 minutes the supernatant was discarded and the pellet was washed twice with PBS.

For intracellular staining the blood was mixed with BD FACS^TM^ Lysing Solution, twice its volume, diluted 1:10 (BD Biosciences, Erembodegem, Belgium), and incubated for 10 minutes. After a washing step at 500 x g for five minutes using PBS containing 1.25% pooled dog serum, BD FACS^TM^ Permeabilizing Solution 2 was added for 10 minutes according to the description of the manufacturer (BD Biosciences). Ultimately, an additional washing step was performed.

The number of leukocytes was determined in both procedures using a hemocytometer and the cell suspension was adjusted to 2.5 10^5^ leukocytes/50 μL using PBS containing 1.25% pooled dog serum.

### Isolation, permeabilization and fixation of cerebrospinal fluid leukocytes

Immediately after tapping, CSF was aliquoted in two tubes and centrifuged at 200 x g for 10 minutes, as described by Schwartz *et al.*[26].

As described above for blood leukocytes, one CSF aliquot underwent fixation (cell surface TLRs) and the other CSF aliquot underwent permeabilization (intracellular TLRs). Both procedures were performed as described for PB with the exception of the lysing steps.

### Monoclonal antibodies (mAbs) and immunostaining

A study from Burgener *et al.*[25] demonstrated that commercial antibodies against human TLRs cross-react with canine TLRs. According to this study, human antibodies were chosen and listed in Table 2. In addition, antibodies against cell surface antigens were used to identify leukocyte subclasses, such as lymphocytes (CD3^+^ or CD21^+^), polymorphonuclear cells (CD11a^+^/CD11b^+^) and monocytes (CD14^+^), (see Table 2). The secondary antibody was an F(ab’)_2_-fragment specific RPE-labeled goat-anti-mouse IgG antibody (Dianova, Hamburg, Germany) (1:200 dilution).

**Table 2:** Monoclonal antibodies

**Specificity**	**Name**	**Clone**	**Provider**	**Dilution**
CD282/TLR2*	mouse anti human CD282	TL2.1	Serotec	1:26
CD283/TLR3*	mouse anti human CD283	TLR3.7	Serotec	1:26
CD 284/TLR4*	mouse anti human CD284	HTA125	Serotec	1:26
CD289/TLR5*	mouse anti human CD289	85B152.5	Acris	1:26
CD289/TLR9*	mouse anti human CD289	5 G5	Serotec	1:26
CD3	mouse anti dog CD3	CA17.2A12	Serotec	1:300
CD11a	mouse anti dog CD11a	CA11.4D3	Serotec	1:300
CD11b	mouse anti dog CD11b	CA16.3E10	Serotec	1:6
CD14*	mouse anti human CD14^§^	TÜK4	Dako	1:15
CD21	mouse anti canine CD21^§^	CA2.1D6	Serotec	1:6
IgG1	mouse IgG1 negative control	W3/25	Serotec	1:6
IgG2a	mouse IgG2a control^§^	PPV-04	ImmunoTools	1:6

*Reacts with canine species; ^§^RPE-Conjugated.

Negative controls consisted of isotype-matched primary antibodies (see Table 2) and cell suspensions stained with the secondary antibody alone.

Incubation was performed for 30 minutes at 4°C under light protection. The washing steps were performed with PBS with 1.25% of canine pooled serum in order to prevent unspecific Fc-receptor binding of mAbs.

### Flow cytometry

Samples were analyzed using a standard FACSCalibur™ flow cytometer and the BD CellQuest™ Pro Version 5.2.1 software (Becton Dickinson, Heidelberg, Germany).

**Figure 1:**
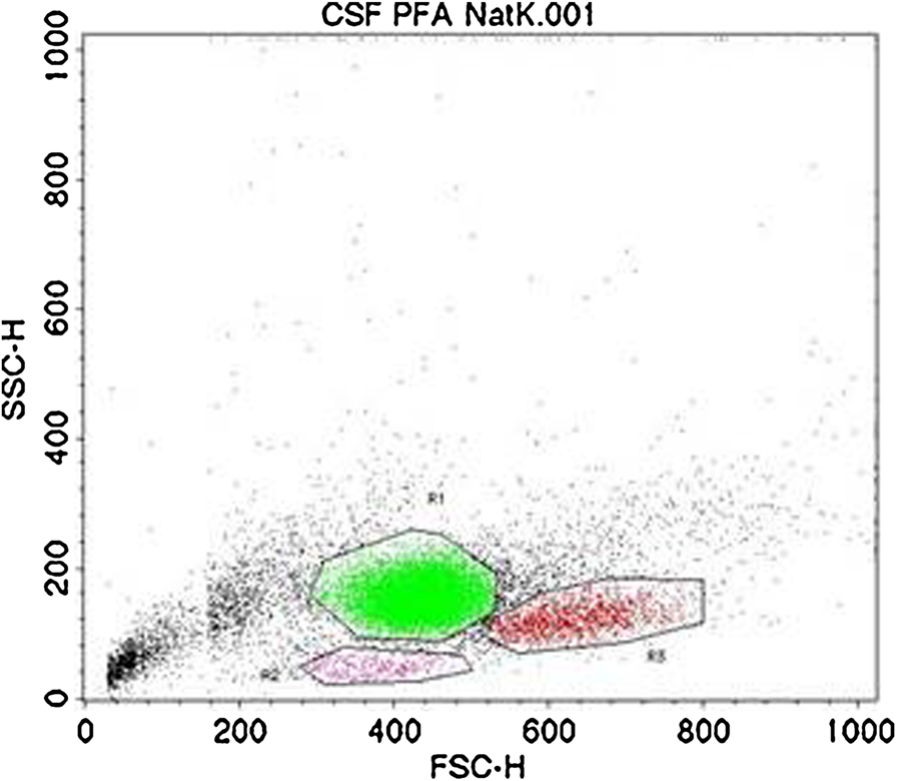
**Population of CSF leukocytes: granulocytes (R1, green), lymphocytes (R2, pink) and monocytes (R3, red).**

Leukocyte populations (Figure 1) were gated according to light scatter properties and CD expression into lymphocytes, monocytes and polymorphonuclear cells (PMNs), as previously described [27].

All events in CSF samples and a minimum of 10,000 events in blood samples were collected.

### Statistical analysis

The percentage of positive cells and the mean fluorescence intensity (MFI) of each group were used for statistical analysis using a commercial statistical program (GraphPad Prism 5.0, GraphPad Software, San Diego, CA, USA). The Wilcoxon rank sum test and the Kruskal–Wallis one-way analysis of variance were applied for comparison of the results deriving from the different groups. Statistical significance was set at the 5% level (*P* <0.05).

## Results

### Expression of TLRs on CSF and PB leukocytes

**Figure 2:**
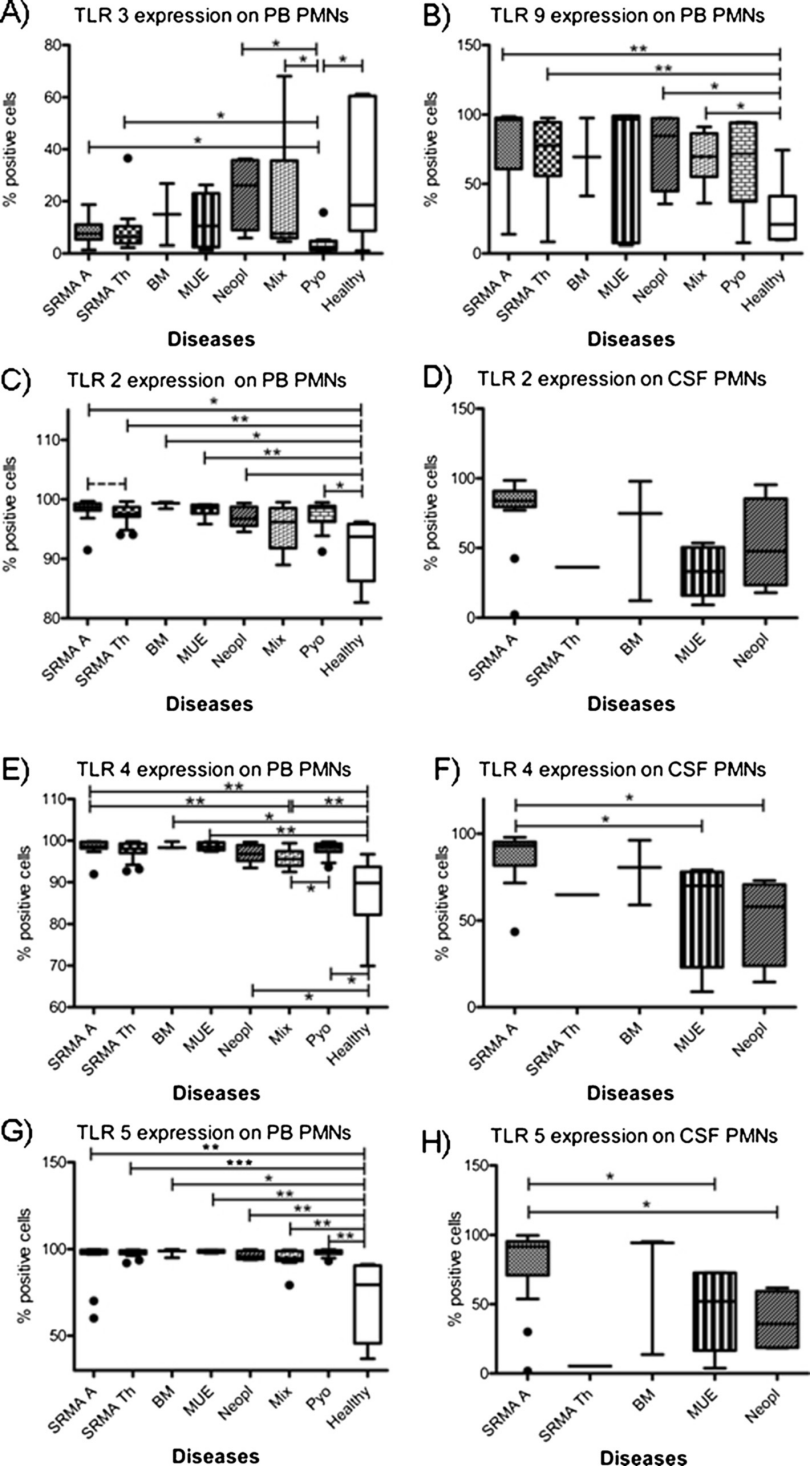
**Percentage of TLR positive PMNs in different disease categories. ** Boxes contain values from the 1^st^ to the 3^rd^ quartile, lines inside boxes indicate median values, endpoints of vertical lines display the 5^th^ to 95^th^ percentile and · represent the outliers. Asterisks indicate statistically significant differences (* *P* <0.05; ** *P* <0.01; *** *P* <0.005). BM, bacterial meningitis; CSF, cerebrospinal fluid; Healthy, healthy dogs; Mix, miscellaneous diseases of the nervous system; MUE, meningoencephalitis of unknown etiology; PB, peripheral blood; PMNs, polymorphonuclear cells; Pyo, pyogenic infection; SRMA, steroid-responsive meningitis-arteritis; SRMA A, SRMA Acute; SRMA Th, SRMA under therapy; TLR, Toll-like receptor.

**Figure 3:**
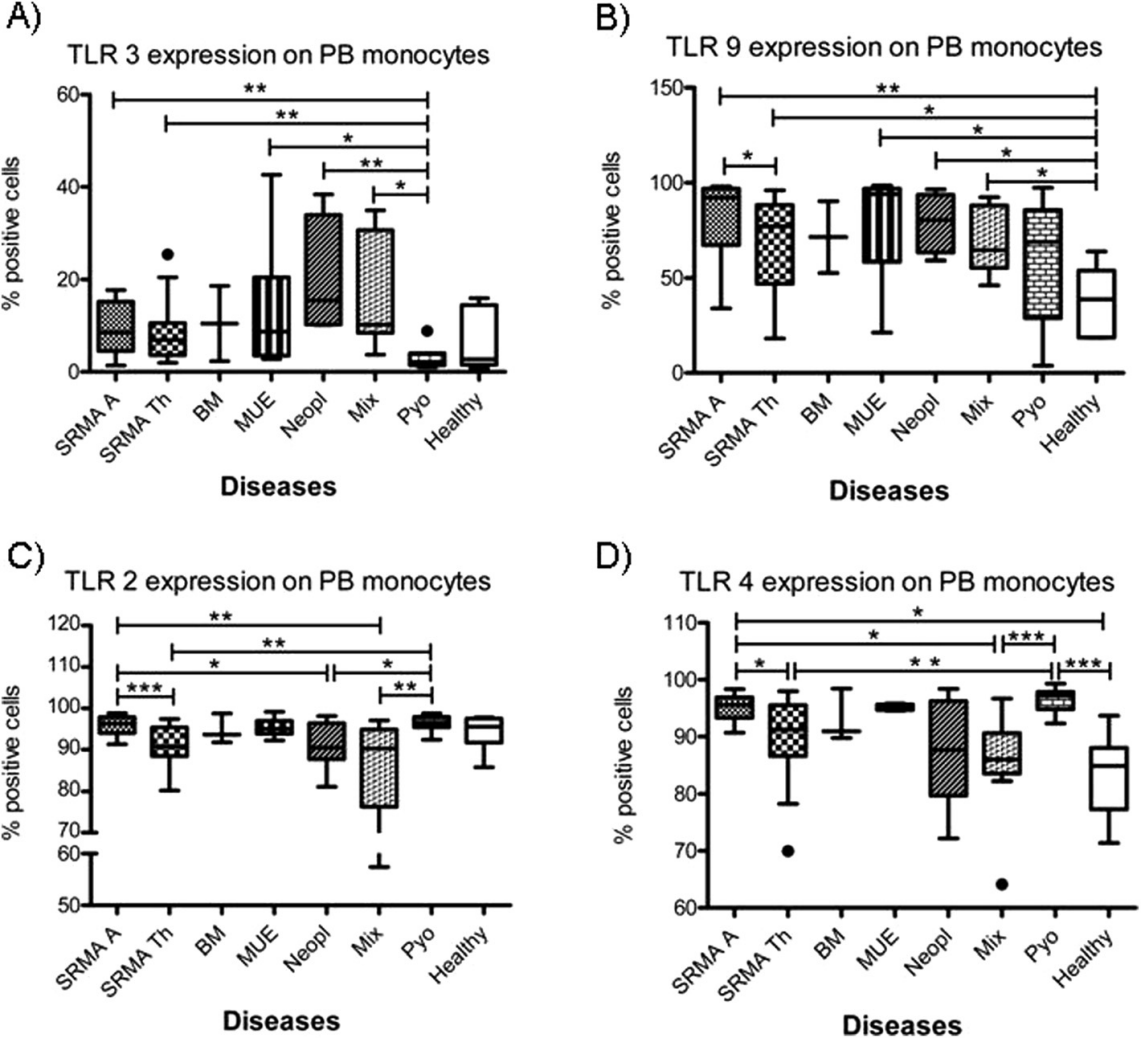
**Precentage of TLR positive monocytes in different disease categories. ** Boxes contain values from the 1^st^ to the 3^rd^ quartile, lines inside boxes indicate median values, endpoints of vertical lines display the 5^th^ to 95^th^ percentile and · represents the outliers. Asterisks indicate statistically significant differences (* *P* <0.05; ** *P* <0.01; *** *P* <0.005). BM, bacterial meningitis; CSF, cerebrospinal fluid; Healthy, healthy dogs; Mix, miscellaneous diseases of the nervous system; MUE, meningoencephalitis of unknown etiology; PB, peripheral blood; PMNs, polymorphonuclear cells; Pyo, pyogenic infection; SRMA, steroid-responsive meningitis-arteritis; SRMA A, SRMA Acute; SRMA Th, SRMA under therapy; TLR, Toll-like receptor.

**Figure 4:**
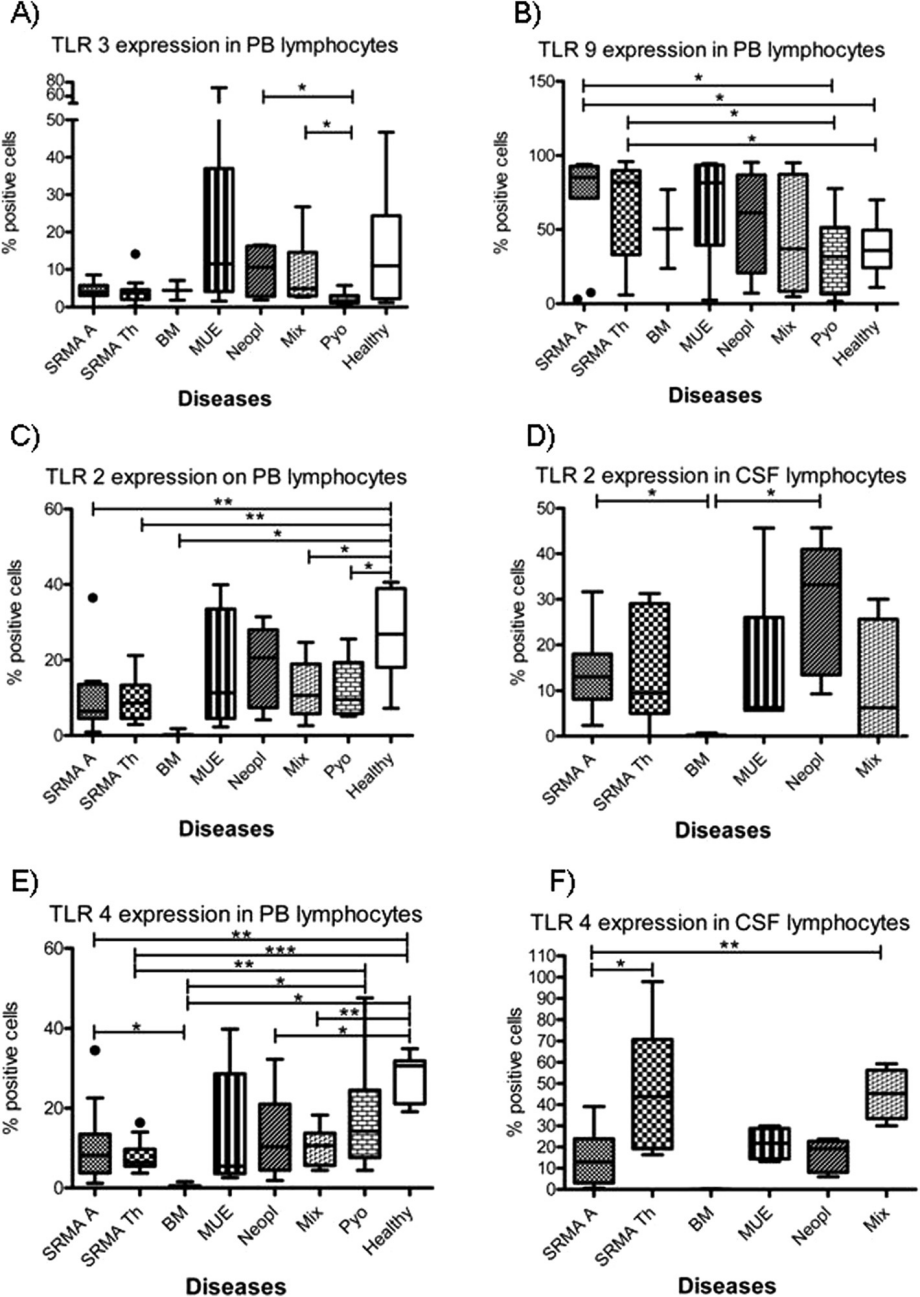
**Percentage of TLR positive lymphocytes in different disease categories. ** Boxes contain values from the 1^st^ to the 3^rd^ quartile, lines inside boxes indicate median values, endpoints of vertical lines display the 5^th^ to 95^th^ percentile and · represents the outliers. Asterisks indicate statistically significant differences (* *P* <0.05; ** *P* <0.01; *** *P* <0.005) BM, bacterial meningitis; CSF, cerebrospinal fluid; Healthy, healthy dogs; Mix, miscellaneous diseases of the nervous system; MUE, meningoencephalitis of unknown etiology; PB, peripheral blood; PMNs, polymorphonuclear cells; Pyo, pyogenic infection; SRMA, steroid-responsive meningitis-arteritis; SRMA A, SRMA Acute; SRMA Th, SRMA under therapy; TLR, Toll-like receptor.

The expression of intracellular and surface TLRs on CSF and PB leukocytes of untreated dogs affected with SRMA are summarized in Table 3; results are given as the percent of positive cells. Statistically relevant results among the different leukocyte subsets and disease categories are shown in Figures 2, 3 and 4.

**Table 3:** **Expression of TLRs in/on leukocytes in SRMA (percentage of positive cells, median and 25% to 75% range) **

**TLRs**	**SRMA A**	**significant differences (*P*** **<0.05)**	**tendencies**
**CSF PMNs**			
TLR3	2.4 (1.5 to 3.7)	none	Neopl↑
TLR9	94.1 (53.8 to 99.0)	none	
TLR2	83.8 (79.6 to 90.9)	none	
TLR4	93.1 (81.7 to 95.3)	MUE↓	
		Neopl↓	
TLR5	91.6 (70.9 to 95.3)	MUE↓	
		Neopl↓	
**CSF monocytes**			
TLR3	14.4 (3.7 to 30.5)	none	
TLR9	85.0 (48.0 to 95.0)	none	MUE↓
TLR2	87.0 (78.9 to 94.3)	none	MUE↓
TLR4	82.6 (74.5 to 93.2)	none	
TLR5	83.3 (62.8 to 94.1)	none	
**CSF lymphocytes**			
TLR3	2.9 (1.0 to 11.6)	none	SRMA Th↑
TLR9	2.9 (18.8 to 1.5)	none	mix↓
TLR2	13.0 (8.1 to 18.0)	BM↓	
		MUE↓	
TLR4	13.0 (3.0 to 23.9)	SRMA Th↑	
		BM↓	
		Mix↑	
TLR5	11.1 (3.7 to 18.8)	none	BM↓
**PB PMNs**			
TLR3	7.5 (5.3 to 11)	Pyo↓	Neopl↑
			Healthy↑
TLR9	96.3 (60.8 to 97.7)	Healthy↓	Mix↓
TLR2	98.7 (98.1 to 99.3)	Healthy↓	SRMA Th↓
			Mix↓
TLR4	98.6 (98.1 to 99.7)	Healthy↓	
		Mix↓	
TLR5	99.0 (97.4 to 99.5)	Healthy↓	
**PB monocytes**			
TLR3	8.6 (4.5. to 15.21)	Pyo↓	Neopl↑
			Healthy↓
TLR9	92.1 (67.2 to 96.9)	SRMA Th↓	Pyo↓
		Healthy↓	
TLR2	96.3 (94.0 to 97.9)	SRMA Th↓	
		Neopl↓	
		Mix↓	
TLR4	95.6 (93.3 to 96.9)	SRMA Th↓	Neopl↓
		Mix↓	
		Healthy↓	
TLR5	95.3 (92.3 to 97.4)	Healthy↓	Mix↓
**PB lymphocytes**			
TLR3	4.0 (3.1 to 5.8)	none	
TLR9	85.2 (71.2 to 92.8)	none	Pyo↓
			Healthy↓
TLR2	6.4 (4.5 to 13.6)	BM↓	MUE↑
		Healthy↑	Neopl↑
TLR4	8.2 (3.8 to 13.5)	BM↓	Pyo↑
		Healthy↑	
TLR5	6.125 (4.4 to 18.7)	none	BM↓
			Healthy↑

BM, bacterial meningitis; Healthy, healthy dogs; Mix, miscellaneous diseases of the nervous system; MUE, meningoencephalitis of unknown etiology; PMNs, polymorphonuclear cells; Pyo, pyogenic infection; SRMA, steroid-responsive meningitis-arteritis; SRMA A, SRMA acute; SRMA Th, SRMA under therapy; TLR, Toll-like receptor.

(↓) lower or (↑) higher values in comparison to SRMA A.

### Expression of TLRs on PMNs

Generally, all groups with diseased dogs displayed a high percentage of TLR2, TLR4 and TLR5 positive PB PMNs in comparison to healthy dogs.

In SRMA dogs under treatment a tendency (*P* = 0.0669) was noted towards a decreased expression of TLR2 on PMNs (median 97.5%; range 97.1 to 98.7%) in comparison to dogs with the acute form of SRMA (median 98.7%; range 98.1 to 99.3%) (see Figure 2C). In CSF samples, no significant differences among the groups were detected. However, the highest values of TLR2 positive PMNs were seen in dogs in the acute form of SRMA (SRMA A, median 83.8%; range 79.6 to 90.9%) (see Figure 2D).

SRMA dogs showed higher expression (*P* = 0.0023) of TLR4 positive PB PMNs (SRMA A, median 98.6%; range 98.1 to 99.7%) in comparison to healthy controls (Healthy, median 89.8%; range 82.5 to 93.7%) and to dogs affected with miscellaneous diseases of the nervous system (Mix, median 95.6%; range 94 to 97.5%) (*P* = 0.0095) (see Figure 2E). In CSF, the highest values of TLR4 positive PMNs were found in dogs in the acute stage of SRMA (median 93.1%; range 81.7 to 95.3%) with significant differences (*P* <0.05) to cases with encephalitides of unknown etiology (MUE, median 67%; range 23.1 to 77.9%) and with CNS neoplasia (Neopl, median 58.1%; range 24 to 70.7%) (see Figure 2F).

All groups of diseased animals had significantly higher percentages of TLR5 positive PB PMNs (*P* <0.05) in comparison to healthy animals (Healthy, median 79.5%; range 45.7 to 90.6%) (see Figure 2G). In CSF higher percentage of TLR5 positive PMNs (*P* <0.05) was found in SRMA A (median 91.6%; range 70.9 to 95.3%) in comparison with MUE (median 52.1%; range 16.6 to 72.6%) and Neopl (median 35.8%; range 18.7 to 59.3%) (see Figure 2H).

Dogs from SRMA A (median 7.5%; range 5.2 to 11%), SRMA Th (median 6.3%; range 3.9 to 10.4%), Neopl (median 26.1%; range 8.9 to 35.8%) and Mix (median 7.5%; range 8.9 to 35.8%) groups showed a higher percentage of PMNs positive for TLR3 (*P* <0.05) in comparison to dogs with pyogenic infections (Pyo, median 2.4%; range 1.3 to 4.7%). SRMA Th and Pyo dogs had a significant lower (*P* <0.05) percentage of TLR3 positive PMNs in comparison to healthy dogs (see Figure 2A). No significant differences were found between the groups examined for expression of TLR3 positive PMNs in CSF. However, a lower percentage of TLR3 positive PMNs was observed in SRMA A (median 2.4%; range 1.5 to 3.7%) in comparison to Neopl (median 10.5 5%; range 4.3 to 14.8%).

Healthy dogs had the lowest values of TLR9 positive PB PMNs (Healthy, median 20.82%; range 10.1 to 41.2%) in comparison to all other groups (*P* <0.05), with the exception of MUE and Pyo (see Figure 2B). The highest values of TLR9 positive PMNs were detected in SRMA A (median 96.3%; range 60.8 to 97.7%). The groups did not differ statistically regarding TLR9 positive PMNs in CSF.

In general, the fluorescence expression intensity did not differ significantly among the groups examined. An exception was the expression intensity of TLR3 on PB PMNs, SRMA A had a lower TLR3 MFI in comparison to Pyo (*P* = 0.0149) and Healthy (*P* = 0.0077).

### Expression of TLRs on monocytes

Dogs with SRMA, similar to dogs with pyogenic infections, were characterized by high values of TLR4 positive PB monocytes. In SRMA dogs, the percentage of TLR2, TLR4 and TLR 9 positive PB monocytes decreased after therapy.

The highest percentages of TLR2 positive monocytes were found in dogs with pyogenic infections (Pyo, median 96.6%; range 95.4 to 97.9%) and untreated SRMA dogs (SRMA A, median 96.3%; range 94 to 97.9%), being significantly higher than in SRMA Th (median 90.7%; range 88.4 to 95.3%; *P* <0.01), Neopl (median 90.5%; range 87.7 to 96.4%; *P* <0.05) and Mix (median 90.3%; range 76.3 to 94.9%; *P* <0.01) (see Figure 3C). The expression of TLR2 positive monocytes in CSF did not statistically differ among the groups; however, a tendency in SRMA A to have a higher percentage of TLR2 positive monocytes (median 87%; range 78.9 to 94.3%) in comparison with MUE (median 26.2%; range 15.7 to 62.4%) was observed.

Higher percentages of TLR4 positive PB monocytes were found in untreated SRMA dogs (SRMA A, median 95.6%; range 93.3 to 96.9%; *P* = 0.0032) and in dogs with pyogenic infections (Pyo, median 97.2%; range 94.8 to 97.9%; *P* = 0.0015) in comparison to control group (Healthy, median 84.8%; range 77.3 to 88.0%). SRMA dogs under therapy showed a decrease in TLR4 positive PB monocytes (SRMA Th, median 91.2%; range 86.6 to 95.5%; *P* = 0.019) in comparison to the untreated dogs (see Figure 3D). The expression of TLR4 positive monocytes in CSF did not statistically differ among the groups.

Similar to SRMA Th, MUE and Pyo, untreated SRMA dogs showed higher percentage of TLR5 positive PB monocytes (SRMA A, median 95.3%; range 92.3 to 97.4%; *P* = 0.0233) in comparison to healthy dogs (Healthy, median 77.6%; range 27.4 to 91.6%). The expression of TLR5 positive monocytes in CSF did not statistically differ among the groups.

Dogs affected with SRMA showed higher percentages of TLR3 positive PB monocytes (SRMA A, median 8.6%, range 4.5 to 15.21%; *P* = 0.0073) in comparison to dogs affected with pyogenic infections (Pyo, median 2.1%; range 1.4 to 4%). Similar percentages have been found in the remaining groups, with the exception of the healthy controls (see Figure 3A). The expression of TLR3 positive monocytes in CSF did not statistically differ among the groups.

Untreated dogs with SRMA showed higher (*P* = 0.003) percentage of TLR9 positive PB monocytes (SRMA A, median 92.13%; range 67.2 to 96.9%) in comparison to healthy dogs (Healthy, median 38.8%; range 18.7 to 54%). Similar values were found also in the remaining groups. In dogs with SRMA the expression of TLR9 positive PB monocytes statistically decreased (*P* = 0.0499) under therapy (SRMA Th median 77.1%; range 47.1 to 88.4%) (see Figure 3B). The expression of TLR9 positive monocytes in CSF did not statistically differ among the groups.

The fluorescence expression intensity did not differ significantly among the examined groups; however, a tendency of SRMA A in expressing higher TLR9 MFI in PB PMNs in comparison to Pyo and Healthy was observed.

### Expression of TLRs on lymphocytes

Generally, lymphocytes of SRMA dogs were characterized by a decreased percentage of TLR2 and TLR4 positive cells in PB and decreased TLR4 expression in CSF in comparison to other diseases, whereas TLR9 was highly expressed in PB.

Dogs affected with SRMA showed lower percentages (*P* = 0.0094) of TLR2 positive PB lymphocytes (SRMA A, median 6.2%; range 1.5 to 13.6%) in comparison to healthy dogs (Healthy, median 26.8%; range 18.1 to 38.9%). Similar findings were seen in the other groups, with the exception of MUE and Neopl. The lowest values were found in dogs with bacterial meningoencephalitis (BM, median 0.2%; range 1.8 to 0.1%), being statistically lower than those found in SRMA A (*P* = 0.0197) (see Figure 4C). SRMA A and SRMA Th dogs also showed lower fluorescence intensity values than Pyo and Healthy, being statistically relevant only for SRMA Th (*P* <0.01). SRMA A had a higher percentage of TLR2 positive lymphocytes in CSF (median 13%; range 8.1 to 18%) than BM (median 0.2%; range 0.1 to 0.7%) (see Figure 4D). Similar differences were observed between these two groups in MFI values.

SRMA A (*P* = 0.0076) and the remaining groups (except MUE and Pyo) had a lower percentage of TLR4 positive PB lymphocytes than Healthy (median 30.6%; range 21.1 to 31.9%) (see Figure 4E). SRMA Th had lower MFI values than Pyo (*P* = 0.0002) and Healthy (*P* = 0.004). Dogs with untreated SRMA displayed statistically lower (*P* <0.01) percentage of TLR4 positive lymphocytes in CSF (SRMA A, median 13%; range 3 to 23.9%) compared to SRMA dogs under therapy (SRMA Th, median 43.8%; range 19.2 to 70.7%;) and dogs with miscellaneous diseases of the nervous system (Mix, median 45.3%; range 33.4 to 56.2%) (see Figure 4F). In SRMA A also the MFI values were statistically lower (*P* = 0.0004) in comparison to SRMA Th.

The percentage of TLR5 positive lymphocytes in PB and CSF did not differ among the groups. However, significant differences among the groups were found in the fluorescence expression intensity of TLR5 in PB lymphocytes. Dogs from both the SRMA A and SRMA Th groups showed lower MFI values in comparison to dogs affected with pyogenic diseases (*P* = 0.02 and *P* = 0.003, respectively). Additionally, there was a tendency for SRMA A towards lower MFI values in comparison to healthy dogs, but this difference was statistically relevant only for SRMA Th (*P* = 0.0056).

The percentage of TLR3 positive lymphocytes in PB in dogs affected with SRMA did not differ from the other groups (see Figure 4A). Also statistically relevant differences in TLR3 expression on CSF lymphocytes were not found. Nevertheless, a tendency of a lower percentage of TLR3 positive CSF lymphocytes in SRMA A (median 2.9%; range 1 to 11.6%) comparing to SRMA Th (median 19.35%; range 4.4 to 38.4%) was detected.

A higher percentage (*P* <0.05) of TLR9 positive PB lymphocytes in untreated SRMA dogs (SRMA A, median 85.24%; range 71.2 to 92.8%) and under treatment (SRMA Th, median 81.9%, range 33 to 89%) were found in comparison to dogs with pyogenic diseases (Pyo, median 31.63%; range 6.6 to 51.4%) and healthy dogs (Healthy, median 35.8%; range 24.2 to 49.6%) (see Figure 4B). The percentage of TLR9 positive lymphocytes in CSF did not statistically differ among the investigated groups.

## Discussion

In recent decades, studies on the etiopathogenesis of SRMA mostly focused on the role of lymphocytes [3,21,28,29]. Indeed, for many years the adaptive immune system has been believed to play the most important role in triggering an inappropriate immune response. However, more recently, the innate immune system aroused much interest for its ability to modify the adaptive immune response, particularly in autoimmunity and immune-mediated diseases [30,31]. TLRs are important components of the innate immune system: their ability to initiate and propagate inflammation protects the organism from infectious diseases [32]. On the other hand, an excessive activation of these receptors may lead to immune disorders [10,30,33,34]. The ambivalent role of these receptors makes them interesting candidates for immune pathological studies in SRMA patients, especially because CSF cells can be studied by systematic flow cytometric studies in this large animal model. The activation profile of TLRs in SRMA was tested to support the hypothesis that these receptors are stimulated by infectious antigens or endogenous proteins (self-antigens). It was hypothesized that they are key factors for the initiation of the inflammatory process and provide an indirect hint of the etiology of the disease. Therefore, the expression of TLRs in dogs affected with SRMA was measured and compared to infectious diseases or other neurological conditions.

The hypothesis that SRMA is maintained by a continuous bacterial infection had to be rejected by the current study. The comparison of TLRs expression profiles of SRMA dogs with dogs affected with bacterial/pyogenic infection failed to show clear similarities between the two groups. In addition, the clear response to long-term treatment with glucocorticosteroids does not support a suspected classical bacterial infection [35-37]. However, triggering of an autoimmune reaction by bacteria cannot be ruled out completely in SRMA.

Indeed, TLR4 was statistically more frequently expressed on monocytes of dogs with untreated SRMA and dogs with pyogenic infections. TLR4 recognizes not only lipopolysaccharides, but also some endogenous ligands, such as heat shock proteins (HSP60, HSP70), fibronectin, hyaluronic acid, fibrinogen and heparan sulfate [32]. In the current study, the triggering factor for increased TLR4 expression, a self or non-self antigen, was not examined. A recent study showed that HSP 70 is elevated in SRMA [38]. Therefore, it seems to be very likely that the triggering protein in SRMA might derive from a self-antigen such as the HSP 70.

The role of TLR4 in human patients with sepsis, but also in non-infectious diseases, such as inflammatory bowel disease and rheumatoid arthritis, were studied [39]. In the canine patient the role of TLR4 during sepsis and the related systemic inflammatory response syndrome has still not been investigated. However, a potential role of TLR4 in dogs with osteoarthritis [40] and chronic enteropathies [20] was proven.

The role of TLR4 for neutrophil recruitment into the CNS was demonstrated in a murine model of systemic inflammation [41]. Similar mechanisms might lead to the invasion of neutrophils into the subarachnoidal space in SRMA and explain the exorbitant neutrophilic pleocytosis in acute cases. A recent study on human large vessel vasculitides suggested that TLR4 is causing transmural panarteritis [16]. Clinical and histopathological findings in dogs affected with SRMA include neutrophilic leukocytosis, neutrophilic pleocytosis and systemic vasculitis. Treatment with glucocorticosteroids reduces these pathological processes and the expression of TLR4 on monocytes declines significantly contemporarily (*P* = 0.019). These findings strongly suggest that TLR4 plays an important role in triggering the described pathological findings in SRMA. Additionally, it opens the discussion for new treatment modalities, such as anti-TLR4 antibodies and TLR4 antagonist; some compounds from the latter class are already under clinical trials for treatment of sepsis in human patients [39,42].

TLR9 seems to be constantly increased on PB leukocytes in almost every disease examined in the current study. However, patients with SRMA and dogs affected with meningoencephalitides of unknown etiology showed the highest expression of TLR9, suggesting a potential role of this TLR in inflammatory CNS diseases with a possible autoimmune component. TLR9 is primarily involved in the recognition of bacterial DNA [32]. In human medicine, TLR9 also seems to play an important role in class-switching to pathogenic autoantibody production in systemic autoimmunity [11], suggesting the possibility that an autoimmune reaction can maintain the inflammatory process in SRMA. However, the role of TLR9 inducing autoimmunity is controversially discussed. TLR9 can have a more regulatory function [43] or enhance pathologic processes [19] in the same animal model of multiple sclerosis. Further studies are needed to elucidate the role of TLR9 in SRMA.

Dendritic cells, once activated by TLR4 and TLR9, produce interleukin-23 (IL-23), which subsequently activate CD4^+^ T cells. The last are known to shift towards Th17-differentiation under the effect of IL-6 and transforming growth factor beta 1 (TGFβ_1_) [30,44]. This relatively new class of T helper cells is believed to be involved in triggering aberrant immune responses and recruiting neutrophils [45,46]. A recent study of our research group showed the concomitant increased intrathecal production of IL-6 and TGF beta-1 in dogs affected with SRMA [47], suggesting a new hypothesis: that the aberrant immune response in SRMA might be associated with Th17 cells maintaining the autoimmune reaction.

Although most TLR responses lead to inflammation, there are studies suggesting an important role of TLRs in homeostasis [43,48]. The role of certain TLRs has been validated in different human diseases resulting in a wide research area focusing on possible new treatment strategies [42]. In case of such multivalent receptors, the great challenge is to reduce the unnecessary inflammation without affecting regulatory functions of TLRs. For example, many efforts attempt to find partial TLR4 agonists, rather than antagonists, and some compounds are already currently available for human use [39,42]. So far in companion animals, the interest has been limited to TLR ligands for developing new vaccines [49], but considering the rapid progress in human medicine, a similar breakthrough is expected soon in veterinary medicine. SRMA would be an ideal model to study such treatment strategies.

Limitations of this study include the absence of true controls, the number of patients in some groups and the variation of the time of sampling in the course of the disease. We tried to overcome the limitations comparing a broad spectrum of different neurological diseases. Including only a few cases of a relatively uncommon disease in dogs, bacterial meningoencephalitis, might be the reason TLRs in CSF samples were not statistically comparable. We tried to overcome this limitation by including dogs with pyogenic infections not affecting the nervous system, since SRMA is considered to be a systemic inflammatory disorder [23].

## Conclusion

We suggest that TLRs are involved in different aspects of the pathogenesis of SRMA. This study supports the hypothesis that an infectious agent can only trigger the disease. SRMA itself seems to be maintained by multiple alterations of the immune system resulting in an autoimmune disease, TLRs, such as TLR4 and TLR9, might act as receptors maintaining the inflammation.

## Abbreviations

BM: Bacterial meningitis; CD: Cluster of differentiation; CNS: Central nervous system; CSF: Cerebrospinal fluid; DAMPs: Damage associated molecular pattern molecules; EDTA: Ethylene diamine tetraacetic acid; HSP: Heat shock proteins; IL: Interleukin; mAb: Monoclonal antibody; MFI: Mean fluorescence intensity; Mix: Miscellaneous non-inflammatory neurological diseases; MRI: Magnetic resonance imaging; MUE: Meningoencephalitis of unknown etiology; Neopl: CNS neoplasia; PAMPs: Pathogen-associated molecular patterns; PB: Peripheral blood; PBS: Phosphate-buffered saline; PMNs: Polymorphonuclear cells; Pyo: Pyogenic infection; rSpear: Spearman’s rank correlation coefficient; SRMA: Steroid-responsive meningitis-arteritis; TGF: Transforming growth factor; Th17: T helper 17; TLR: Toll-like receptor.

## Competing interests

The authors declare that they have no competing interests.

## Authors’ contributions

AT designed and supervised the study. AM performed the experiments and analyzed the data. RC gave substantial contributions to acquisition, analysis and interpretation of the data. AM drafted the manuscript and all authors contributed to the critical revision of the manuscript for important intellectual content and have read and approved the final version.
